# Obesity, Inflammation, and Severe Asthma: an Update

**DOI:** 10.1007/s11882-021-01024-9

**Published:** 2021-12-18

**Authors:** Varun Sharma, Douglas C. Cowan

**Affiliations:** 1 Institute of Infection, Immunity and Inflammation, University of Glasgow, Glasgow, UK; 2 Glasgow Royal Infirmary, Glasgow, UK

**Keywords:** Asthma, Obesity, Inflammation, Weight loss

## Abstract

**Purpose of Review:**

Obesity-associated difficult asthma continues to be a substantial problem and, despite a move to address treatable traits affecting asthma morbidity and mortality, it remains poorly understood with limited phenotype-specific treatments. The complex association between asthma, obesity, and inflammation is highlighted and recent advances in treatment options explored.

**Recent Findings:**

Obesity negatively impacts asthma outcomes and has a causal link in the pathogenesis of adult-onset asthma. Imbalance in the adipose organ found in obesity favours a pro-inflammatory state both systemically and in airways. Obesity may impact currently available asthma biomarkers, and obesity-associated asthma specific biomarkers are needed. Whilst surgical weight loss interventions are associated with improvements in asthma control and quality of life, evidence for pragmatic conservative options are sparse. Innovative approaches tackling obesity-mediated airway inflammation may provide novel therapies.

**Summary:**

The immunopathological mechanisms underlying obesity-associated asthma require further research that may lead to novel therapeutic options for this disease. However, weight loss appears to be effective in improving asthma in this cohort and focus is also needed on non-surgical treatments applicable in the real-world setting.

## Introduction

Asthma remains common, is characterised by variable symptoms and airflow obstruction, and is associated with a significant global health burden [1]. The UK continues to rank highly in obesity prevalence internationally (8.1% of UK 15–19-year-olds with obesity in 2015 [2]), has one of the highest asthma mortality rates in Europe, and has disproportionately increased asthma mortality in young people compared to other European countries [2, 3]; for example, the UK asthma mortality rate for ages 20–24 per 100,000 in 2016 was 0.30 (95% CI 0.28–0.33), compared to the same age-specific population in Italy with mortality rate 0.03 (95% CI 0.02–0.05) [2]. Asthma associated with obesity is a particular challenge, being less steroid-responsive and linked to poorer control, increased medication use, longer hospital stays, poorer quality of life, and greater severity of disease [4–9]. Moreover, other obesity-associated comorbidities such as obstructive sleep apnoea syndrome (OSAS) appear to worsen outcomes [10]. In this review, we provide an update on associations between obesity and asthma, current understanding of potential immunological mechanisms, recent interventional trials, and possible future therapies.

## Obesity

Over 650 million adults are obese worldwide [11]. The rising obesity epidemic over the past few decades has led to increased pressure on healthcare resources and rising morbidity and mortality from obesity-associated disease. Obesity impacts on outcomes in many of the most common lung diseases including chronic obstructive pulmonary disease (COPD), asthma, OSAS, and obesity hypoventilation syndrome (OHS) [12]. The effect of obesity in pulmonary disorders is multifaceted. Obesity alters chest wall dynamics with mass effect directly affecting thoracic mechanics, an integral component of the process of ventilation. Raised body mass index (BMI) is associated with increased airflow limitation; for example, a recent Danish cross-sectional study showed OR 3.1 (95% CI 1.97–4.78, *p* < 0.001) and OR 1.7 (95% CI 1.08–2.68, *p* = 0.023) for airway obstruction in overweight and obese subjects respectively [13].

Airway closure is associated with gas trapping and hyperinflation and is one of two elements in FEV_1_ reduction (alongside airway narrowing). In asthma, raised BMI has recently been shown to affect airway closure independently of asthma control [14]. A further study has shown that raised BMI, but not waist circumference (WC, a marker of central obesity), worsens airway closure in people with asthma (*p* = 0.01), suggesting the effect is not entirely related to altered chest mechanics [15].

Dietary changes associated with obesity, such as high-sugar, high-fat, and low-fibre intake, are also associated with increased airway inflammation, at least in murine models [16, 17].

## Asthma and Obesity: Cause and Effect

The association between obesity and asthma has historically been considered uni-directional; people with severe asthma become less active and deconditioned, weight increases, and increased usage of oral corticosteroids (OCS) further aggravates the weight gain cycle. Whilst this cohort of patients undeniably exists, increasing evidence supports the causal link between raised BMI, asthma, and poor asthma control (Table 1 [18–37]). Cluster cohort studies show a now widely recognised female-predominant obese severe asthma phenotype [38–40], particularly affecting peri- and post-menopausal women [37]. However, a large cross-sectional US study has shown higher levels of oestradiol (OR 0.43 for current asthma, 95% CI 0.23–0.78) and testosterone (OR 0.59 for current asthma, 95% CI 0.37–0.91) appearing to play a protective role in obese asthma [41∙]. The links between obesity and asthma are complex and perhaps best considered to be bi-directional [24]. Poorer asthma control and quality of life, increased corticosteroid use, and emergency service attendance in this patient cohort have been demonstrated in numerous studies worldwide [7, 19, 20, 26, 27, 31].

**Table 1:** Links between obesity and asthma — summary of recent studies

Study	Study population	Study design	Relevant points
Sun et al. [18∙]	Norwegian, ≥ 20 years of age. *N* = 56 105	Mendelian randomisation analysis	OR **1.36** (95% CI 1.10–1.68), **1.49** (95% CI 1.14–1.94) and **1.40** (1.02–1.93) per 4.1 kg/m^2^ BMI increase and “ever asthma,” doctor-diagnosed asthma, and doctor-diagnosed active asthma respectively
Abrahamsen et al. [19]	Norwegian, 16–50-year-old patients with symptomatic asthma. *N* = 326	Cross-sectional	OR_adj_ **2.2** (95% CI 1.2–4.1, *p* < 0.05) for BMI ≥ 30 kg/m^2^ association with poor asthma control
Alves et al. [20]	Brazilian, asthma patients ≥ 18 years of age. *N* = 473	Cross-sectional	OR_adj_ **1.46** (95% CI **0.89**–2.39) for BMI ≥ 30 kg/m^2^ in severe asthma
Souza et al. [21]	Brazilian, patients ≥ 40 years of age. *N* = 1026. Asthmatics, *N* = 116	Cross-sectional	PR_adj_ **2.3**; 95% CI 1.2–4.5 (*p* = 0.01) for overweight and asthma, **3.1**; 95% CI 1.6–6.0 (*p* = 0.001) for obesity and asthma
Park et al. [22]	South Korean, 40–79-year-old patients without asthma. *N* = 459 529	Cohort study, outcome was development of asthma	Multivariable HR **1.23** (95% CI 1.13–1.34) and **1.40** (95% CI 1.32–1.48) for development of asthma with BMI ≥ 30 kg/m^2^ in men and women respectively
Lampalo et al. [23]	Croatian, adult patients, *N* = 302, divided into asthmatic and non-asthmatic groups	Cross-sectional	Increased BMI associated with asthma in women (*p* = 0.002)
Zhu et al. [24]	UK, 16 + years of age. *N* = 457 822	Cross-trait genome-wide association study	OR **1.21** SE 0.04 (*p* = 6.3 × 10^−7^) for causal effect of raised BMI on later-onset asthma
Borna et al. [25]	Sweden, age 16–75 years. *N* = 24,534	Cross-sectional	OR **2.60** (95% CI 1.63–4.13) for current asthma and BMI > 30 kg/m^2^, and OR **2.50** (95% CI 1.61–3.88) for physician-diagnosed asthma and BMI > 30 kg/m^2^
Irani et al. [26]	Lebanon, age 18 + years. *N* = 183	Cross-sectional	OR_adj_ **0.155** (95% CI 0.062–0.389, *p* < 0.001) and **0.131** (95% CI 0.035–0.485, *p* = 0.002) for BMI 25–29.9 kg/m^2^ and ≥ 30 kg/m^2^ respectively (compared to normal BMI) and poor asthma control
Ohta et al. [27]	Japan, age 18 + years. *N* = 421	Cross-sectional	OR **1.05** (95% CI 1.02–1.08, *p* = 0.002) for BMI and asthma exacerbation
Petermann-Rocha et al. [28]	Chile, age 15 + years. *N* = 5499	Cross-sectional	OR **1.13** (95% CI 1.04 – 1.22. *p* < 0.01) for BMI and asthma, OR **1.15** (95% CI 1.06–1.25, *p* < 0.01) for WC
Xu et al. [29]	Multi-national, European ancestry	Mendelian randomisation analysis	OR **1.18** (95% CI 1.11–1.25, *p* = 2 × 10^−8^) per unit increase of BMI on risk of asthma
Solet et al. [30]	Reunion Island, age 18–44 years. *N* = 2419	Cross-sectional	OR **1.52** (95% CI 1.02–2.28) for obesity and suspected asthma
Neffen et al. [31]	Multi-national, Latin American, age 12 + years. *N* = 594	Cross-sectional	OR_adj_ **1.71** (95% CI 1.04–2.84, *p* = 0.036) obesity and uncontrolled asthma
Vandenplas et al. [32∙]	Multi-national, European, adults with occupational asthma. *N* = 162	Cross-sectional	OR **1.98** (95% CI **0.97**–3.97, *p* = **0.056**) for obesity and severe occupational asthma
Aarab et al. [33]	Netherlands, multiple ethnic groups, age 18 + years. *N* = 23,356	Cross-sectional	OR_adj_ **1.07** (95% CI 1.06–1.08) for BMI and adult-onset asthma across all ethnic groups
Lurbet et al. [34]	USA, age 18 + years. *N* = 543 574	Cross-sectional	OR **1.75** (95% CI 1.75–1.76) for obesity with asthma
Klepaker et al. [7]	Norway, age 18–52 years. *N* = 626	Cross-sectional	OR **1.78** (95% CI 1.14–2.80), **1.81** (95% CI 1.03–3.18) for asthma with BMI ≥ 30 kg/m^2^ and higher symptom score and poor asthma control respectively
Tomita et al. [35]	Japan, age 40–64 years. *N* = 9888	Cross-sectional	OR_adj_ **1.92** (95% CI 1.35–2.75, *p* < 0.01), **2.24** (95% CI 1.23–4.09, *p* < 0.01), **1.89** (95% CI 1.30–2.75, *p* < 0.01) and **1.53** (95% CI 1.15–2.03, *p* < 0.01) for asthma in women only and BMI 25–29.9 kg/m^2^, BMI ≥ 30 kg/m^2^, WC ≥ 90 cm and WHt ratio ≥ 0.5 respectively
Santos et al. [36]	Brazil, age 18–45 years. *N* = 60,202	Cross-sectional	OR_adj_ **1.49** (95% CI 1.14–1.96) for asthma and obesity
Matulonga-Diakiese et al. [37]	France, women without asthma at baseline, age 41–68 years. *N* = 67 872	Cohort study, outcome was development of asthma	HR_adj_ **1.91** (95% CI 1.00–3.66) and **2.08** (95%CI 1.07–4.06) for overweight/obese peri-menopausal and post-menopausal women respectively and asthma

*Adj* adjusted, *BMI* body mass index, *CI* confidence interval, *HR* hazard ratio, *OR* odds ratio, *PR* prevalence ratio, *SE* standard error, *WC* waist circumference, *WHt* waist-to-height

Obesity has been shown to increase inflammation in people with and without asthma [42∙, 43∙∙]. A large Japanese genome-wide association study (*n* = 9789, 4% with asthma) [43∙∙] found increasing BMI correlates with increased blood neutrophil and eosinophil count (until BMI approximately 40 kg/m^2^ when eosinophil counts levels off). However, another interesting find from this study was that those with an elevated eosinophil level at baseline had a negative association with increasing BMI, suggesting deeper complexity in the relationship between adipose excess and inflammation. A recent European prospective study (*n* = 202) reported an additive effect of asthma and obesity on increased release of pro-inflammatory mediators and airway inflammation as well as modification of the gut, nasal, oral, and lung microbiome, intimately linked with inflammation [42∙]. Use of SPECT/CT scanning in obese-asthma has recently shown increased lung eosinophil uptake compared to healthy BMI counterparts further emphasising the impact of obesity on airway inflammation [44]. The presence of dietary polyunsaturated and saturated fatty acids increase release of inflammatory cytokines during respiratory tract infection which may enhance airway inflammation and therefore impact severity of asthma exacerbation [45].

Raised BMI also impacts airways directly through increased airway hyper-responsiveness in populations with asthma [46], and effects on human airway smooth muscle are exaggerated in the obese female adult population [47].

The relationship between increased adiposity and asthma becomes more complicated as visceral adiposity, independent of BMI or waist circumference, affects asthma-related quality of life [48]. Reasons for this include reduced lung function, and increased effects of related comorbidities such as acid reflux and depression; however, the intricate science of adipose tissue and its relationship to inflammation will undoubtedly be important. Further evidence exists to support the argument that obesity-related comorbidities, particularly presence of the metabolic syndrome, also contribute to poorer asthma outcomes [49]. Central obesity, evidenced by raised waist-to-height ratio, and insulin resistance negatively impact lung function in patients with asthma [50].

## Adipose Tissue and Inflammation

Adipose tissue is categorised as “brown” (adipocytes with high mitochondria count and numerous small lipid droplets), “white” (few mitochondria and a large single lipid droplet), or “brown-like” or “beige” (intermediate mitochondria and lipid droplet count compared to white or brown adipocytes) [51] and accumulates to form brown adipose tissue (BAT), white adipose tissue (WAT), and beige adipose tissue respectively. A more recently recognised but less well understood peri-vascular adipose tissue (PVAT) has been documented (Table 2) [52, 53]. Adipose “tissue” forms a complex endocrine organ intimately involved in inflammation homeostasis and is not merely an inert energy reservoir as previously thought. Excessive WAT, seen in obesity, plays a pro-inflammatory role, though healthy BAT acts to regulate these negative effects. However, a process of “browning” WAT (i.e., production of beige adipose tissue) can negate the pro-inflammatory effects of WAT also [53]. As such, BAT and beige adipose tissue can be thought of as “protective” against pro-inflammatory states, whilst excess WAT enhances inflammation. Furthermore, BAT activity is reduced in obesity (so called “whitening” of BAT), with increased dysfunctional mitochondria suggested as a factor [54, 55]. Further understanding is needed in this area; however, there is a relationship between the immune system and adipose tissue with activated macrophages and CD8 + T cells identified as important players [56]. Upregulation of BAT or browning of WAT may have a role to counteract the low-level systemic inflammation caused in obesity. Following bariatric surgery, adipose tissue composition can significantly change with increased BAT and increased browning of WAT associated with an improved inflammatory state post-operatively [57].

**Table 2:** Summary of adipose tissue anatomical location and function

Component	Location	Function
BAT [52, 53]	Predominantly interscapular and subscapular regions, supraclavicular, neck, peri-renal, mediastinal	Primarily non-shivering thermogenesis, oxidative metabolism. Evidence of autocrine and paracrine signals promoting BAT recruitment. Evidence of endocrine signals, e.g., secretion of insulin-like growth factor 1 improving glycaemic control, counteracting WAT-induced inflammation and pro-inflammatory adipokine secretion
WAT [52, 53]	Subcutaneous and abdominal, including visceral adipose deposits	Energy storage. Endocrine functions — secretion of leptin, adiponectin, IL-6, TNFα
Beige adipose tissue [52, 53]	Within WAT, predominantly subcutaneous	Unclear but can display functions of BAT and WAT (thermogenesis, energy storage)
PVAT [52]	Peri-vascular	Unclear but involvement with regulating vascular tone and thermogenesis. Can appear similar to BAT or WAT

*BAT* brown adipose tissue, *IGF* insulin-like growth factor, *IL* interleukin, *PVAT* peri-vascular adipose tissue, *TNF* tumour necrosis factor, *WAT* white adipose tissue

Key cytokines produced from adipose tissue have been identified that may be useful as novel biomarkers in management of obesity and obesity-associated inflammatory disease [58]. A summary of effects is provided in Table 3 [59–62].

**Table 3:** Key adipokines — normal function and effects in obesity

Peptide	Function	Effect in obesity
Leptin [59]	Hypothalamic regulation of feeding behaviour	Increased. Resistance of feeding-behaviour effects. Pro-inflammatory cytokine production and activation of monocytes and macrophages
Adiponectin [59, 60]	Insulin, glucose and fatty acid homeostasis. Anti-inflammatory and immunomodulatory actions	Reduced secretion
IL-6 [59, 60]	Insulin, fatty acid homeostasis and effects on energy expenditure. Can act as pro- or anti-inflammatory	Increased secretion, increased pro-inflammatory effects
TNFα [59, 60]	Mediates tumour necrosis. Pro-inflammatory. Increased lipolysis and decreased insulin signalling	Increased secretion
Resistin [59, 60]	Unclear. Increases insulin resistance	Increased secretion. Increased pro-inflammatory cytokines, increases pulmonary inflammation
IL-10 [60–62]	Immunomodulatory effects. Reduces pro-inflammatory cytokine synthesis and decreases macrophage activity. Reduces release of reactive oxygen species and cytotoxic T-cell response	Conflicting reports of both high and low levels in obesity compared to healthy BMI. One suggested explanation for high levels in obesity is of a homeostatic attempt to inhibit other pro-inflammatory adipokines. The presence of metabolic syndrome associated with reduced IL-10, irrespective of the presence of obesity
CCL2 [59, 60]	Immunomodulatory effects in adipose tissue	Increased pro-inflammatory effects
Chemerin [59]	Immunomodulatory effects, pro-inflammatory but has potential anti-inflammatory effects. Role in adipocyte metabolism	Increased pro-inflammatory effects

*BMI* body mass index, *CCL2* CC-chemokine ligand 2, *IL* interleukin, *TNF* tumour necrosis factor

Increased leptin and IL-6 levels in asthma patients have recently been identified in several studies, strengthening the link of these pro-inflammatory cytokines with airway inflammation in asthma [63–65].

## Obese-Asthma and a Need for Biomarkers

Routinely used markers of allergy and eosinophilic inflammation in asthma include serum total IgE, fractional exhaled nitric oxide (FeNO), and serum and (where available) sputum eosinophils. Evidence suggests that obesity may have direct effects on these biomarkers, and this has implications for accuracy of phenotyping and determination of suitability for currently available biologic treatments [66, 67]. Previous studies have shown that increased BMI is negatively correlated with FeNO and this may be related to the increased airway oxidative stress associated with obesity [68, 69]. More recently, Winnica et al. [70∙∙] found that respiration at a mitochondrial level varies in obese-asthma, when compared to obese controls, healthy BMI-asthma, and healthy BMI controls, with diminished cellular nitric oxide (NO) bioavailability in this cohort resulting in reduced FeNO.

One US multi-centre study [71∙∙] of 652 adults with mild to moderate asthma revealed poor correlations between increased BMI and the four measured type 2 (T2) inflammatory markers (FeNO, IgE, sputum, and serum eosinophils). FeNO levels were reduced in obese-asthma and, despite > 85% of participants having positive skin prick testing, IgE levels were also reduced in this group compared to their healthy BMI counterparts. Serum eosinophils, total IgE, and FeNO did not correlate with sputum eosinophilia levels in obesity-associated asthma. The differences seen compared to leaner individuals is perhaps related not only to the increased oxidative stress caused by obesity but also to direct disruption of eosinophil recruitment and survival caused by adipocytokines [71∙∙].

Whilst much research has focussed on biomarkers and treatment options for asthma with T2-high inflammation, it remains widely appreciated that further research is needed to elucidate potential biomarkers and treatment options for the non-T2 and/or T2-low endotypes and this may be particularly relevant to obesity-associated asthma [72–76].

## Management of Obesity-Associated Asthma: Scope of the Problem

In an era of precision medicine, there remains a dearth of specific treatments for obesity-associated severe asthma. Standard current treatment options comprise escalation of historically available asthma therapies, i.e., inhaled corticosteroids (ICS), long-acting beta-agonists (LABA), long-acting muscarinic antagonist (LAMA), leukotriene receptor antagonists, oral theophylline, macrolides and OCS, and more recently available biologic treatments (if eligible). Identification and treatment of treatable traits have more recently been encouraged in the management of asthma; however, there remains a paucity of interventions for T2-low or non-T2 severe obese-asthma. Beyond advising healthy living and referring to local weight management services, there are limited choices for dealing with this “treatable” trait.

Despite the wealth of evidence showing positive impacts of bariatric surgery, access to this remains poor for a variety of reasons [77], and surgical risk is not insignificant. Conservative interventions are needed for those in whom surgical options are not appropriate or desired.

Advanced obese-asthma therapies are limited, and there is a need for precision biomarkers that can be used to target specific interventions.

## Weight Reduction Strategies in Asthma: Lifestyle and Surgical Interventions

Further recent studies have evaluated the impact of weight loss strategies on obesity-associated asthma, and in particular the effects of bariatric surgery (Table 4 [78–86]). Surgical techniques, such as Roux-en-Y gastric bypass and sleeve gastrectomy, are associated with reduction in both systemic and airway pro-inflammatory markers, improvement in lung function, asthma control and quality of life scores (including ACT, ACS, AQLQ), and a reduction in treatment burden [78–81]. Some evidence suggests that asthma remission may also result, but this needs to be confirmed [82, 83]. Whilst these studies have limitations including small sample size and open-label design, the available evidence is that bariatric surgery may lead to improvements in outcomes in obese asthma, many of which may be sustained in the mid-to-long term. In contrast, another study by Forno et al.[81] showed a lack of improvement, following bariatric surgery in obesity-associated asthma with concomitant metabolic disease (defined as three out of five of the following: abdominal obesity, raised triglycerides, low HDL, hypertension, and hyperglycaemia). This implies that this phenotype of obese asthma is particularly difficult to treat, and that weight loss alone may not be effective in these patients; a multifaceted, individualised, and targeted approach may be necessary.

**Table 4:** Summary of surgical and non-surgical intervention trials

Study	Population	Intervention	Design	Follow-up duration	Outcome(s)	Result
Baltieri et al. [78]	Brazil. Age 18–65-year-old women, BMI ≥ 35 kg/m^2^, respiratory clinician diagnosed asthma. *N* = 18	Bariatric surgery — RYGB	Open-label prospective cohort study, single-centre	12 months after surgery	(1) Systemic and sputum inflammatory markers — adiponectin, IL-6, IL-8, leptin, resistin, TNF-α, CRP (2) ACT	Reduced systemic IL-8, CRP, leptin, TNF-α (*p* value 0.002, 0.003, 0.001, 0.007 respectively). Increased systemic IL-6 (*p* value 0.004). Reduced pulmonary TNF- α (*p* value < 0.001) ACT increased from 18 (range 5–23) to 25 (range 24–25), *p* value < 0.0001
Santos et al. [79]	Portugal. Age 18 + years. Physician diagnosed obese asthmatics (*n* = 8), obese non-asthmatics (*n* = 18)	Bariatric surgery – gastric bypass or vertical gastrectomy	Open-label, prospective longitudinal study, single-centre	6–9 months after surgery	(1) Pulmonary function tests (2) CARAT, ALQ (3) Asthma medication usage	Improvement in lung function in both groups, with no statistically sig difference Improved CARAT score for lower airways (4.2 ± 4.4, *p* value = 0.027) and improved ALQ score (8.1 ± 5.6, *p* value = 0.017) Decrease in asthma treatment step (− 1.8 ± 1.0, *p* value = 0.017)
Guerron et al. [80]	USA. Age 18 + years. Obese patients on at least one asthma medication (*n* = 751)	Bariatric surgery — RYGB, sleeve gastrectomy, adjustable gastric banding, duodenal switch	Retrospective analysis	3 years after surgery	Asthma medication usage	Adjusted rate ratios of count of asthma medications 0.73 (95% CI 0.66–0.80, *p* < 0.0001) and 0.54 (95% CI 0.45–0.65, *p* < 0.0001) at 30 days post-op and 3 years post-op respectively
Forno et al. [81]	USA. Age 18 + years with self-reported asthma diagnosis (*n* = 555). Comparing those with and without metabolic syndrome	Bariatric surgery — RYGB, laparoscopic adjustable band, sleeve gastrectomy, other	Prospective observational cohort study, multi-centre	6 years after surgery	ACT	Proportion of metabolic syndrome negative obese asthma patients with an ACT > 19 (i.e., adequate control) increased from 58 to 78% at 60 months. Outcomes for metabolic syndrome positive patients poorer; however, many results not statistically significant
Wazir et al. [82]	UK. Age 18–68. Primarily study of obese patients with T2DM. *N* = 121 in total, *n* = 70 with asthma	Bariatric surgery – sleeve gastrectomy, adjustable gastric band, one anastomosis gastric bypass, RYGB	Retrospective analysis	Two years after surgery	Primary outcomes related to T2DM remission Secondary outcomes included remission of obesity-related comorbidities including asthma	18 (25.7%) of patients with asthma had remission; however, definition of remission not given, and asthma-related outcomes not specifically analysed
Samuel et al. [83]	UK. Adults divided into morbidly obese (BMI 40–49.9 kg/m^2^), super-obese (BMI 50–59.9 kg/m^2^) and super-super-obese (BMI > 60 kg/m^2^). *N* = 64 asthmatics (353 patients in total)	Bariatric surgery — laparoscopic RYGB, laparoscopic adjustable band, laparoscopic sleeve gastrectomy	Retrospective analysis	Two years after surgery	Secondary outcome included mid-term remission of obesity-related comorbidities including asthma (however criteria for asthma remission not evident)	In the super-morbidly obese that underwent RYGB, 6 (5.9%) had remission of asthma (*p* value = 0.014)
Grandi Silva et al. [84]	Brazil. Physician diagnosed asthma in women aged 30–60 with BMI ≥ 35 and < 40 kg/m^2^. *N* = 42. Analysis divided into two groups: those that lost > 5% body weight and those that lost < 5% body weight	Diet and exercise programs (3 months) with psychology support	Prospective, non-controlled study	3 months	Primary outcome — improvement of DH and EFL Secondary outcomes include ACQ, AQLQ, airway inflammatory markers (FeNO, IL-2, IL-4, IL-5, IL-10)	Improved DH during submaximal exercise and increased time to onset of DH and EFL in > 5% weight group > 5% weight group had > 0.5 clinically significant improvement in both ACQ and AQLQ, and statistically significant improvement in most AQLQ domains (except environmental stimuli) compared to < 5% weight group > 5% weight group: •Reduced FeNO (− 7.94 ± 12.24 ppb, *p* value = 0.04) •Reduced pro-inflammatory interleukins (IL-2 − 25.33 ± 72.55, and IL-4 − 3.13 ± 7.72, *p* values 0.02 and 0.05 respectively) •Increased anti-inflammatory interleukin (IL-10 41.83 ± 63.44, *p* value 0.003)
Lang et al. [85]	USA. Age 12–25 years. Overweight/obese patients with uncontrolled asthma. *N* = 98	Omega-3 fatty acid (n3 polyunsaturated fatty acid) supplementation	Randomised, double-blind, placebo-controlled, parallel design study, multicentre	24 weeks	Primary outcome — change in ACQ at 6 months Secondary outcomes — ACT, lung function and inflammatory biomarkers	No significant difference in ACQ, ACT, lung function or biomarkers
Holguin et al. [86∙∙]	USA. Age 18–66 years. Physician-diagnosed asthma. BMI ≥ 30 kg/m^2^, FeNO ≤ 30 ppb	L-citrulline (15 g/day) supplementation	Open-label pilot, proof-of-concept study, multicentre	2 weeks	Primary outcome — rise in FeNO Secondary outcome included ACQ	Increased FeNO (4.2 ppb, 95% CI 1.8 to 6.7, *p* value = 0.001) Decreased ACQ (− 0.46, 95% CI − 0.67 to − 0.27, *p* = 0.001)

*ACQ* Asthma Control Questionnaire, *ACT* asthma control test, *ALQ* asthma life quality, *AQLQ* Asthma Quality of Life Questionnaire, *BMI* body mass index, *CARAT* control of allergic rhinitis and asthma test, *CI* confidence interval, *CRP* C-reactive protein, *DH* dynamic hyperinflation, *EFL* expiratory flow limitation, *FeNO* fractional exhaled nitric oxide, *IL* interleukin, *RYGB* Roux-en-Y gastric bypass, *T2DM* type 2 diabetes mellitus, *TNF* tumour necrosis factor

An open-label, prospective study [84] utilising diet and a structured exercise program for three months in 51 obese women with physician-diagnosed moderate to severe asthma showed that, alongside improvements in lung volumes and airflow on exertion, loss of > 5% of body weight had a favourable impact on asthma biomarkers (FeNO), systemic biomarkers (reduced IL-2, IL-4, increased IL-10), and patient-centred outcomes (ACQ, AQLQ).

The impact of dietary factors on asthma is another area that is receiving interest. For example, high-fat and low-fibre intake have been linked to increasing airway inflammation [87]. Whilst robust evidence for treatments in this area is lacking, there is potential for future research. A pilot study of 41 obese adults with poorly controlled asthma demonstrated a decrease in ACQ, despite an increase in FeNO, with oral administration of 15 g/day L-citrulline supplements after 2 weeks [86∙∙]. The authors suggest that obesity and asthma cause uncoupling of airway epithelium-bound nitric oxide synthase (NOS) causing reduced FeNO in this cohort, and that the increased FeNO seen post-supplementation is due to L-citrulline-mediated recoupling of NOS. This increase in FeNO might have suggested a deleterious effect on asthma outcomes; however, the improved ACQ implies otherwise, further highlighting the complexity of the obesity-inflammation relationship and a need for accurate obese-asthma specific biomarkers. The mean ACQ reduction of − 0.46, whilst statistically significant, failed to meet the minimal clinically important difference of 0.5 [88], but nevertheless, this proof-of-concept trial paves the way for a further suitably powered study. Conversely a double-blind, multicentre, randomised, placebo-controlled trial [85] in adolescent obese asthma did not show any difference in asthma outcomes including biomarkers or lung function with omega-3 fatty acid supplementation although it is possible that higher doses and/or longer course of treatment may have led to more favourable effects.

Further research is needed to clarify the complex mechanisms that underlying the links between obesity and dietary-intake and inflammation in asthma. More evidence from randomised, controlled trials for weight loss in obese-asthma is needed, with pragmatic conservative strategies applicable to the real-world setting.

## Potential Treatments

Whilst targeted therapies may be lacking, pursuing obesity-induced inflammation and repurposing current widely used anti-diabetic drugs for weight loss in obese asthma may pose a viable treatment option.

Recent randomised control trials in obese and healthy-BMI adults with asthma (*n* = 127, *n* = 23 for two trials [89]) have contributed to the understanding of the interplay between airway inflammation and excessive dietary fatty acid and carbohydrate intake. Nucleotide-binding domain leucine-rich repeat and pyrin domain containing receptor 3 (NLRP3)-mediated airway inflammation was observed in healthy BMI individuals with asthma after over-nutrition, and enhanced NLRP3 inflammatory effects were identified in obese asthma participants, including higher levels of IL-5, IL-1β, and sputum neutrophils. Findings suggest that targeting the NLRP3 inflammasome may yield a potential treatment in obese asthma [90].

Anti-diabetic medications that cause weight loss include metformin and glucagon-like peptide 1 agonists/receptor agonists. Neither of these are specific to diabetes, and both are beneficial for non-diabetic weight loss [91–93]. Whilst redirecting these medications to tackle the treatable trait of obesity in asthma might be of benefit, this remains to be proven. Nevertheless, preliminary studies do suggest that both metformin and GLP-1 agonists may improve asthma outcomes, independently of weight loss, perhaps due to underlying anti-inflammatory or immunomodulatory effects, or effects on insulin resistance [94, 95].

## Conclusion

In summary, a better understanding of adipose tissue-associated inflammation and its relationship to asthma is needed. Obesity-associated asthma encompasses several endotypes and phenotypes in addition to the recently recognised adult-onset, female-predominant, non-allergic obese-asthma phenomenon. The presence of metabolic syndrome is associated with increased morbidity and more difficult to treat disease. Currently available biomarkers may be of limited value in obesity-associated asthma, and identification of novel biomarkers is a priority for this difficult-to-treat subset of patients. Though real-world obesity-specific treatments are lacking, trials aiming at weight loss continue to show improvements and novel therapies for obese-asthma may be on the horizon.
